# Simultaneous monitoring of eight human respiratory viruses including SARS-CoV-2 using liquid chromatography-tandem mass spectrometry

**DOI:** 10.1038/s41598-022-16250-y

**Published:** 2022-08-04

**Authors:** Christopher Hodgkins, Laura K. Buckton, Gregory J. Walker, Ben Crossett, Stuart J. Cordwell, Andrea R. Horvath, William D. Rawlinson

**Affiliations:** 1 NSW Health Pathology, Prince of Wales Hospital, Campus Centre Building, 2031 Randwick, NSW Australia; 2 School of Life and Environmental Sciences and Charles Perkins Centre, The University of Sydney, 2006 Sydney, Australia; 3 Virology Research Laboratory, SAViD, Prince of Wales Hospital, 2031 Randwick, NSW Australia; 4 Schools of Medical Sciences, Women’s and Children’s Health, Faculty of Medicine and BABS Faculty of Science, University of New South Wales, 2052 Sydney, NSW Australia; 5 Sydney Mass Spectrometry, The University of Sydney, 2006 Sydney, NSW Australia; 6 Serology and Virology Division (SAViD), NSW Health Pathology, 2031 Randwick, NSW Australia

**Keywords:** Virology, Proteomics, Analytical chemistry

## Abstract

Diagnosis of Severe Acute Respiratory Syndrome Coronavirus 2 (SARS-CoV-2) infection has primarily been achieved using reverse transcriptase polymerase chain reaction (RT-PCR) for acute infection, and serology for prior infection. Assay with RT-PCR provides data on presence or absence of viral RNA, with no information on virus replication competence, infectivity, or virus characterisation. Liquid chromatography-tandem mass spectrometry (LC–MS/MS) is typically not used in clinical virology, despite its potential to provide supplemental data about the presence of viral proteins and thus the potential for replication-competent, transmissible virus. Using the SARS-CoV-2 as a model virus, we developed a fast ‘bottom-up’ proteomics workflow for discovery of target virus peptides using ‘serum-free’ culture conditions, providing high coverage of viral proteins without the need for protein or peptide fractionation techniques. This workflow was then applied to Coronaviruses OC43 and 229E, Influenza A/H1N1 and H3N2, Influenza B, and Respiratory Syncytial Viruses A and B. Finally, we created an LC–MS/MS method for targeted detection of the eight-virus panel in clinical specimens, successfully detecting peptides from the SARS-CoV-2 ORF9B and nucleoprotein in RT-PCR positive samples. The method provides specific detection of respiratory viruses from clinical samples containing moderate viral loads and is an important further step to the use of LC–MS/MS in diagnosis of viral infection.

## Introduction

The COVID-19 pandemic and the subsequent global social and economic disruption have generated great interest in improved laboratory testing for respiratory viruses. Widespread testing for viral infection is a cornerstone of the response to COVID-19, with nearly 2.76 billion Reverse Transcriptase-Polymerase Chain Reaction (RT-PCR) tests for the Severe Acute Respiratory Syndrome Coronavirus 2 (SARS-CoV-2) carried out worldwide from 1st of January, 2020 to 1st of November, 2021^1^. A rapid, sensitive, and accurate test for viral infection is critical in reducing widespread transmission of the virus especially where asymptomatic infections with SARS-CoV-2 frequently occur^2^.

Nucleic Acid Test assays are ubiquitous in clinical virology. Their utility for clinical decision making is limited, however, by the inability to distinguish replication-competent (i.e. live, transmissible) virus from residual viral nucleic acid^3^. In a significant number of cases, SARS-CoV-2 infected patients continue to test positive for viral RNA for days to months after the known window of infection has passed. This significantly impairs decision making around transmission risk and release from quarantine^4^.

Rapid antigen tests have been developed for SARS-CoV-2 and other respiratory viruses, where the antigen target is a viral protein. While providing potential value as a screening tool, reports of the sensitivity and specificity of such tests have been mixed^5,6^ and are most likely manufacturer dependent^7^. Additionally, over time these tests may face lot-to-lot variation, false negatives due to patient-specific antibodies, and other issues already described for antibody-based tests^8^.

Mass spectrometry (MS) has been used as a complementary tool to PCR and other assays in virology research^9^, but so far has not been routinely used in high-throughput clinical testing for viral infection. Liquid chromatography-tandem MS (LC–MS/MS) has become widely used in clinical chemistry for the analysis of small molecules and proteins in a range of biological specimens^10^. Matrix-Assisted-Laser-Desorption-Ionisation coupled to Time-of-Flight (MALDI-TOF) mass spectrometry is used extensively in clinical microbiology to rapidly, accurately, and inexpensively identify the causative agents of bacterial infections^11^. The widespread adoption of MS and the availability of generic sample preparation methods for detection of proteins marked both MALDI-TOF–MS and LC–MS/MS as possible solutions to diagnostic problems in the COVID-19 pandemic. This included early predicted disruptions in supply-chains for RT-PCR reagents and diagnostic problems around transmission.

Direct detection of respiratory viruses by MS in clinical samples had rarely been reported prior to the emergence of SARS-CoV-2^12^, with one report from 2015 describing a 100-fold analytical sensitivity deficit^13^. However, since March 2020 several groups have demonstrated the capability of MS to detect SARS-CoV-2 in small cohorts of clinical specimens including nasopharyngeal swabs^14–16^ and gargle solution^17^ using nanoflow LC, high resolution MS and shotgun proteomics techniques. Studies analysing larger cohorts of SARS-CoV-2 positive and negative nasopharyngeal swabs used validated analytical techniques more common in current routine clinical LC–MS/MS analysis^18^ and provided strong evidence for the extent of correlation between viral RNA and protein concentration in nasopharyngeal swab samples after performing immunocapture of the target protein^19^ or peptide^20,21^ before LC–MS/MS analysis. Later, a consortium of academic laboratories and instrument vendors demonstrated a process to standardise analysis of SARS-CoV-2 proteins across different LC–MS/MS instruments and laboratories but were limited in detecting viral proteins in samples with an RT-PCR cycle threshold (Ct) of greater than 20^22^. MALDI-TOF MS was applied to analysis of clinical samples, targeting either protein^23^ or amplified nucleic acids^24^ with significant discriminating power. However, it has been suggested that direct detection of viral proteins in clinical samples remains beyond the sensitivity of current MALDI-TOF–MS techniques^25^.

As SARS-CoV-2 is predicted to become an endemic virus^26^, there is value in incorporating its detection into a panel of other circulating respiratory viruses. In response, we developed a rapid multiplexed LC–MS/MS test to assess the sensitivity and selectivity of instruments that are currently available in clinical laboratories. Furthermore, in creation of this method, we established a fast bottom-up proteomics workflow for discovery of target virus peptides using ‘serum-free’ culture conditions, providing high coverage of viral proteins without the need for protein or peptide fractionation techniques used when standard culture conditions are employed. Finally, in applying the targeted method to the analysis of 30 SARS-CoV-2 RT-PCR positive nasopharyngeal swab extracts, we provide new evidence for the ORF9B protein as a target for detection of SARS-CoV-2 infection in clinical samples.

## Results

### Serum-free viral culture reduces protein background and improves virus detection

Concentrated virus samples (~ 10^6^ TCID_50_/mL) were generated using cell culture. The SARS-CoV-2 was grown in Vero E6 cells cultured in standard conditions with 2% fetal bovine serum (FBS). High pH fractionation of culture supernatant digests gave adequate coverage of the SARS-CoV-2 proteome in the high bovine serum background from untargeted analysis. There were 44 peptides identified from 7 SARS-CoV-2 proteins (Table 1), representing 0.5% of the 15,813 peptide spectral matches recorded. By contrast, 84.7% of these spectral matches were made to bovine proteins.

**Table 1:** Performance comparison of peptide discovery methods in various cell culture conditions.

SARS-CoV-2 protein	Vero E6 cells cultured in 2% FBS					Vero E6 cells cultured in serum-free conditions		
	High pH fractionation and 12 DDA analyses on Q-Exactive Plus		DDA and SWATH analysis on TripleTOF 6600			DDA and SWATH analysis on TripleTOF 6600		
	# Peptides	% Sequence coverage	# Peptides	% Sequence coverage	% TOF–MS peak area	# Peptides	% Sequence coverage	% TOF–MS peak area
Nucleoprotein (N)	15	38	14	41	0.232	39	80	6.1059
Spike glycoprotein (S)	9	8	6	6	0.0603	23	20	0.7791
Membrane protein (M)	3	17	1	5.4	0.0168	9	29	0.1754
ORF 9b protein	8	88	5	58	0.0445	13	96	2.898
ORF 8 protein	2	13	1	6	0.0166	4	30	0.5121
ORF 7a protein	1	6			0.0009	1	5	0.0024
Replicase polyprotein 1a	6	2						

**Figure 1:**
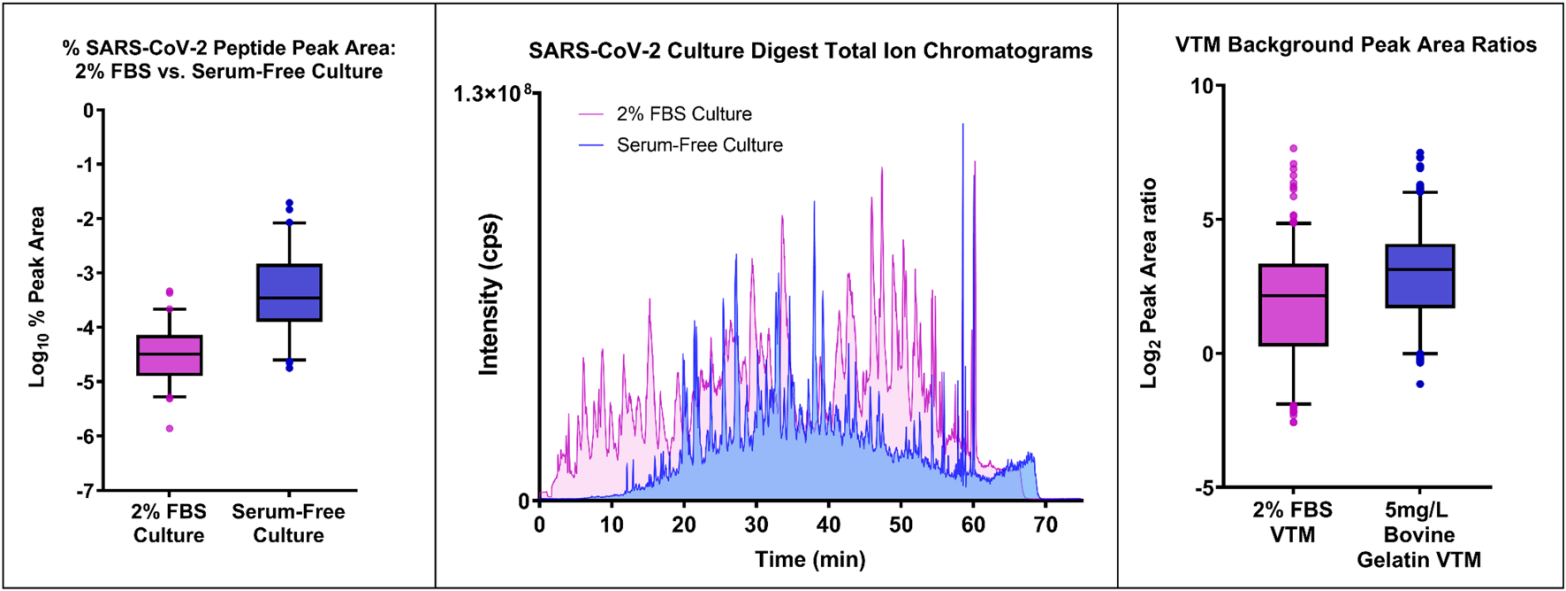
(Left) Box-and-whisker plots of % peak area (TIC-normalised total SWATH fragment peak area) for all detected SARS-CoV-2 peptides in culture digests, showing improved detectability of virus proteins despite overall lower sample loading. (Middle) Overlay of total ion chromatograms (TIC) for SARS-CoV-2 culture digests under standard culturing conditions (2% FBS) and serum-free conditions. Overall higher intensity of signal for FBS culture represents higher total protein loading on the LC–MS. (Right) Box-and-whisker comparison of normalised peak areas for all targeted peptides in spiked samples. The area of each peak in the MRM chromatogram was normalised to the total area under the chromatogram within the detection window.

A baseline for unfractionated analysis of culture supernatant digests was set by a single 2-h analysis on the TripleTOF 6600, resulting in 28 SARS-CoV-2 peptides identified at > 95% confidence. The bovine protein background was then reduced by culturing the virus in Vero E6 cells under serum-free conditions. This greatly improved virus detection from a single run on a TripleTOF 6600, generating higher sequence coverage than the fractionated 2% FBS sample (Table 1), comparable to other published studies^27–29^ using fractionation techniques on viral cultures (Supplementary Table S2). To demonstrate the impact of serum-free conditions on relative virus concentration in culture supernatants, the summed peak area from the best four fragment chromatograms from each SARS-CoV-2 peptide detected during SWATH analysis was expressed as a percentage of the area under the TOF–MS total ion chromatogram (TIC). On average, virus peptides generated 26 times higher signal intensity from when considered as a proportion of the total precursor intensity from each run (Fig. 1). An overlay of the TOF–MS TIC shows the difference in total protein loaded from each sample and the plot of percentage peak areas demonstrates that the total peak area from SARS-CoV-2 peptides is an order of magnitude higher in the serum-free culture compared to the 2% FBS virus culture (Fig. 1). Sequence coverage and peptide numbers from other respiratory virus cultures grown under serum free conditions and assayed by either the nanoflow LC–MS or the alternate microflow LC–MS method (as noted), are presented in Table 2.

**Table 2:** Sequence coverage for all respiratory viruses from analysis of serum-free cultures. Viruses 229E, OC43, RSV-A, RSV-B and SARS-CoV-2 were analysed with a short microflow method, whereas Influenza A and B were analysed with nanoflow LC, resulting in inferior sequence coverage for the former group.

Virus Name	Accession #	Name	% Cov (> 95% conf)	# Peptides (> 95% conf)
Human Coronavirus 229E	sp|P15130|	Nucleoprotein	10	6
Human Coronavirus OC43	sp|P33469|	Nucleoprotein	17	8
Human Coronavirus OC43	sp|Q01455|	Membrane protein	4	2
RSV A	sp|P03421|	Phosphoprotein	8	1
RSV A	sp|P04545|	Matrix M2-1	24	5
RSV A	sp|P0DOE7|	Matrix protein	23	7
RSV B	tr|A0A1P8L2Y4|	Nucleoprotein	2	1
RSV B	tr|A0A1P8L301|	Matrix protein	12	4
RSV B	tr|A0A1P8L3S0|	Fusion glycoprotein F0	2	1
RSV B	tr|A0A1V0E295|	Phosphoprotein	21	4
RSV B	tr|A0A1V0E2A0|	Matrix M2	18	4
SARS-CoV-2	sp|P0DTC2|	Spike glycoprotein	3	4
SARS-CoV-2	sp|P0DTC5|	Membrane protein	5	1
SARS-CoV-2	sp|P0DTC8|	ORF8 protein	6	1
SARS-CoV-2	sp|P0DTC9|	Nucleoprotein	35	14
SARS-CoV-2	sp|P0DTD2|	ORF9b protein	96	11
Influenza A	sp|P03485|	Matrix protein 1	16	3
Influenza A	sp|P03496|	Non-structural protein 1	5	1
Influenza A	tr|E4UHA7|	Nucleoprotein	39	23
Influenza B	tr|A0A126TSZ9|	Hemagglutinin	13	10
Influenza B	tr|A0A126TT08|	Polymerase basic protein 2	3	3
Influenza B	tr|A0A126TT20|	RNA-directed RNA polymerase catalytic subunit	6	4
Influenza B	tr|A0A126TT34|	Nucleoprotein	62	67
Influenza B	tr|A0A126TTR2|	Matrix protein 1	47	16
Influenza B	tr|A0A126TTS5|	Non-structural protein 1	54	25
Influenza B	tr|A0A126TTX5|	Neuraminidase	13	6
Influenza B	tr|A0A126TUL8|	Polymerase acidic protein	8	5

### Transport medium protein source generates differing sensitivity and background interferences

**Figure 2:**
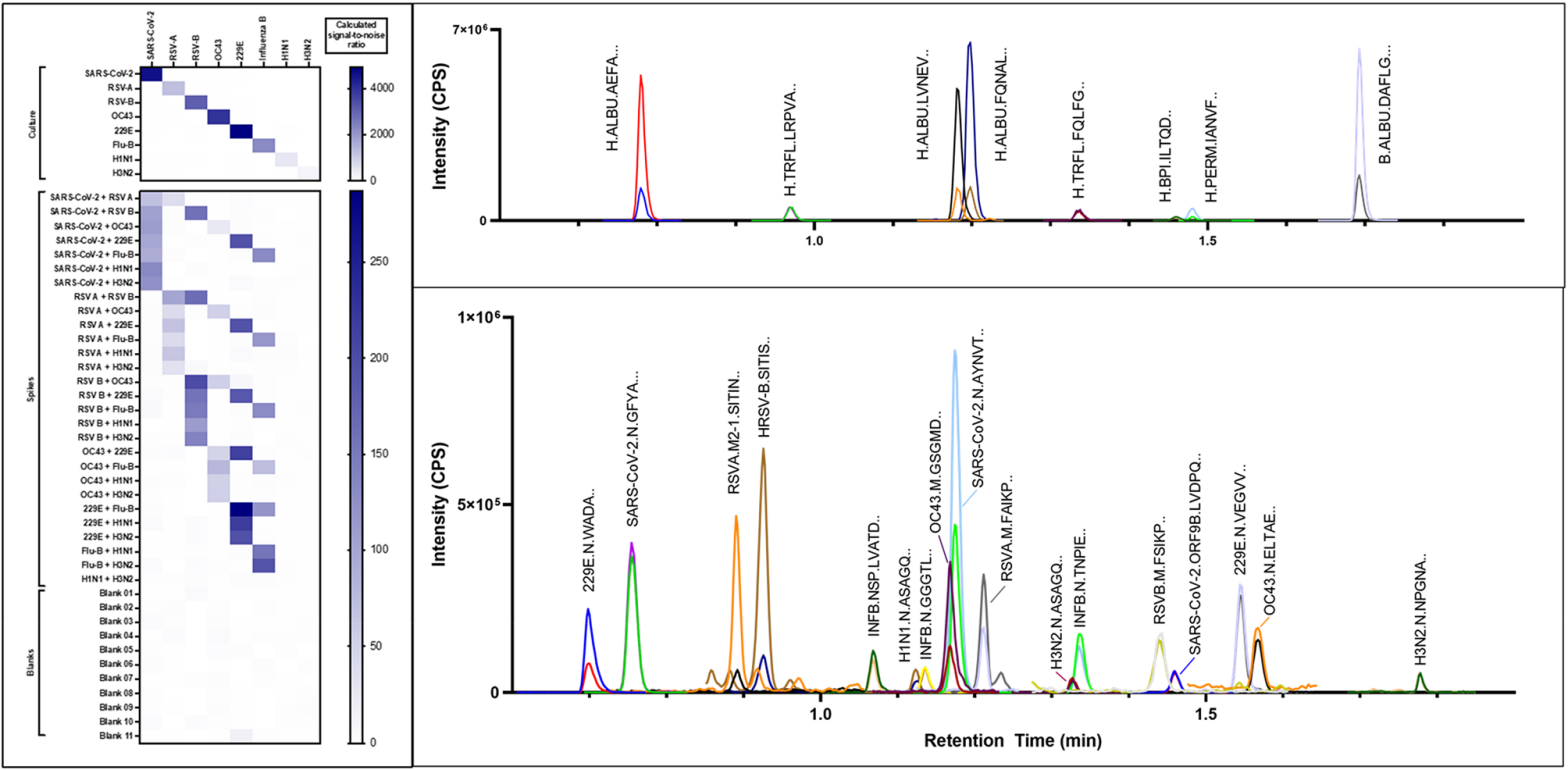
(Left) Heat map of signal-to-noise ratio for combined peak areas of virus spikes (1:9 v/v) versus peak area average of blank samples. (Right) Overlay of MRM chromatograms from human background proteins (upper panel) and virus cultures (lower panel) showing efficient use of MS detection time and minimal concurrency through careful peptide selection and maximising chromatographic separation.

Spiking viral cultures into negative swab extracts stored in VTM containing either FBS or bovine gelatin allowed an additional round of culling based on the presence of interfering signals in MRM traces, which were more prevalent in FBS VTM extracts than in gelatin VTM. For the same relative concentration (*v/v*) of virus spiked into each matrix type, gelatin VTM extracts gave a mean relative response of target peak area to total background area more than 2 times higher than FBS VTM extracts (Fig. 1). Interestingly, one peptide (SNLKPFER from the Spike protein) produced significant overlapping peaks for all MRMs in RT-PCR negative swabs from gelatin VTM but not in FBS VTM swabs, despite being confidently identified (> 99% confidence) in SARS-CoV-2 cultures. As such, this peptide was removed from further analyses. Finally, identical spiked and cultured samples were compared using the original and rapid sample preparation methods and a final peptide list was determined from peptides efficiently liberated during rapid digestion. The two most sensitive and selective MRMs for each peptide were included in the final method (Fig. 2 and Supplementary Table 4).

### Targeted MRM method is specific for each virus

To assess combined method selectivity, we spiked cultures of all eight virus targets into negative nasopharyngeal swabs in all possible paired combinations. Our final targeted MRM method could detect six of the eight target viruses with 100% specificity at a 1:9 (*v/v*) spiking concentration, with no cross-reactivity of viruses or background proteins (Fig. 2). Peptides for H1N1 and H3N2 that were detected in undiluted culture with below average peak areas, potentially indicating comparatively low concentration, fell below the limit of detection at this spiked concentration. Sensitivity of high-flow chromatography, was assessed by scaling flow rate, gradient program and run time on the same column. Similar sensitivity was achieved with all methods; therefore, the flow-split technique was selected as it offered a crucial increase in sample throughput (Supplementary Fig. 1).

**Figure 3:**
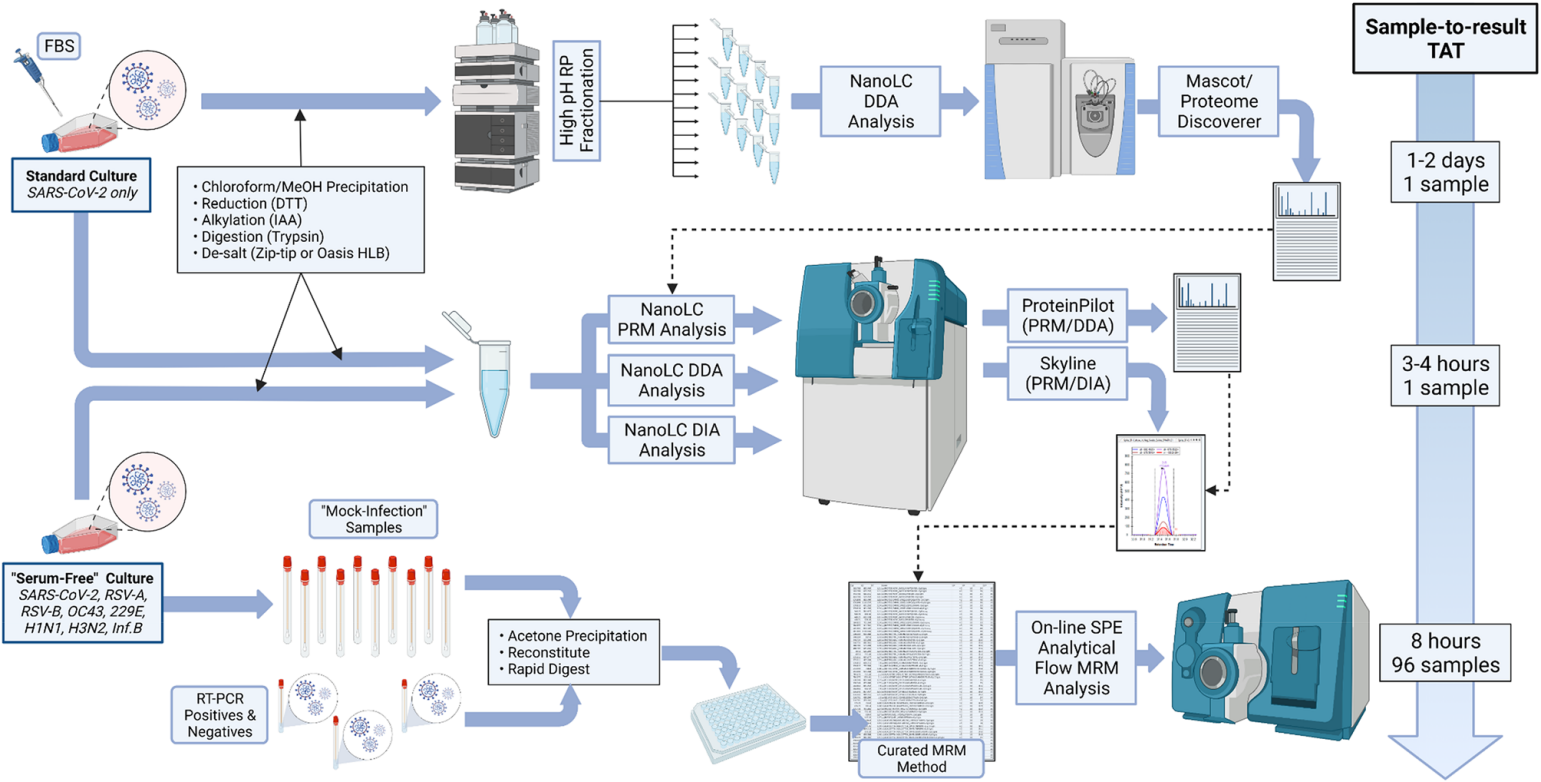
Graphical summary of workflow from viral cultures to rapid targeted MRM method. Use of serum-free culture avoids the need for extensive sample fractionation and use of both data-dependent and data-independent-acquisition methods during discovery phase streamlines peptide selection process. Created with BioRender.com.

The optimised LC–MS/MS method required a similarly rapid sample preparation procedure to ensure a high throughput total sample analysis. A short acetone protein precipitation was incorporated to remove small molecule interferences (particularly phenol red), disrupt virions, and pre-concentrate proteins. Consistent with other published methods, reduction and alkylation steps were omitted as we had deliberately avoided the use of cysteine-containing peptides. Finally, SDC and *n*-propanol denaturants were replaced with a commercial rapid digest buffer to allow the direct injection of the digest on to the trap column without the acid precipitation step required for SDC. Digestion time was reduced from overnight to 1 h for the first set of clinical samples (n = 20) and then increased to 2 h for the second clinical sample set (n = 40). The total workflow from viral culture to targeted MRM method is summarised in Fig. 3.

### Sensitivity of multiplexed virus detection in clinical specimens was consistent with other published methods targeting only SARS-CoV-2

Prior to development of the multi-virus targeted method, an initial cohort of ten clinical specimens RT-PCR positive for SARS-CoV-2 were assayed by a method including only SARS-CoV-2 peptide targets. Cycle thresholds (Ct) values ranged between 17 and 37. Out of ten RT-PCR positive infections, LC–MS/MS detected viral proteins in two clinical specimens that had Ct values of 17 and 19. Virus peptides were not detected in specimens with Ct values greater than 19 (Supplementary Table 5).

Following addition of MRMs for the virus panel to the targeted method, a second cohort of four RSV-A positive and two OC43 positive clinical specimens were subjected to a protein precipitation step and the rapid digestion protocol before analysis. Three out of four RSV-A infections were detected by LC–MS/MS with Ct values of 15, 17, and 22. One clinical specimen with a Ct of 24 was not detectable. The OC43 samples were not detectable by LC–MS/MS, consistent with their relatively high Ct values of 29 and 30 (Supplementary Table 5).

**Figure 4:**
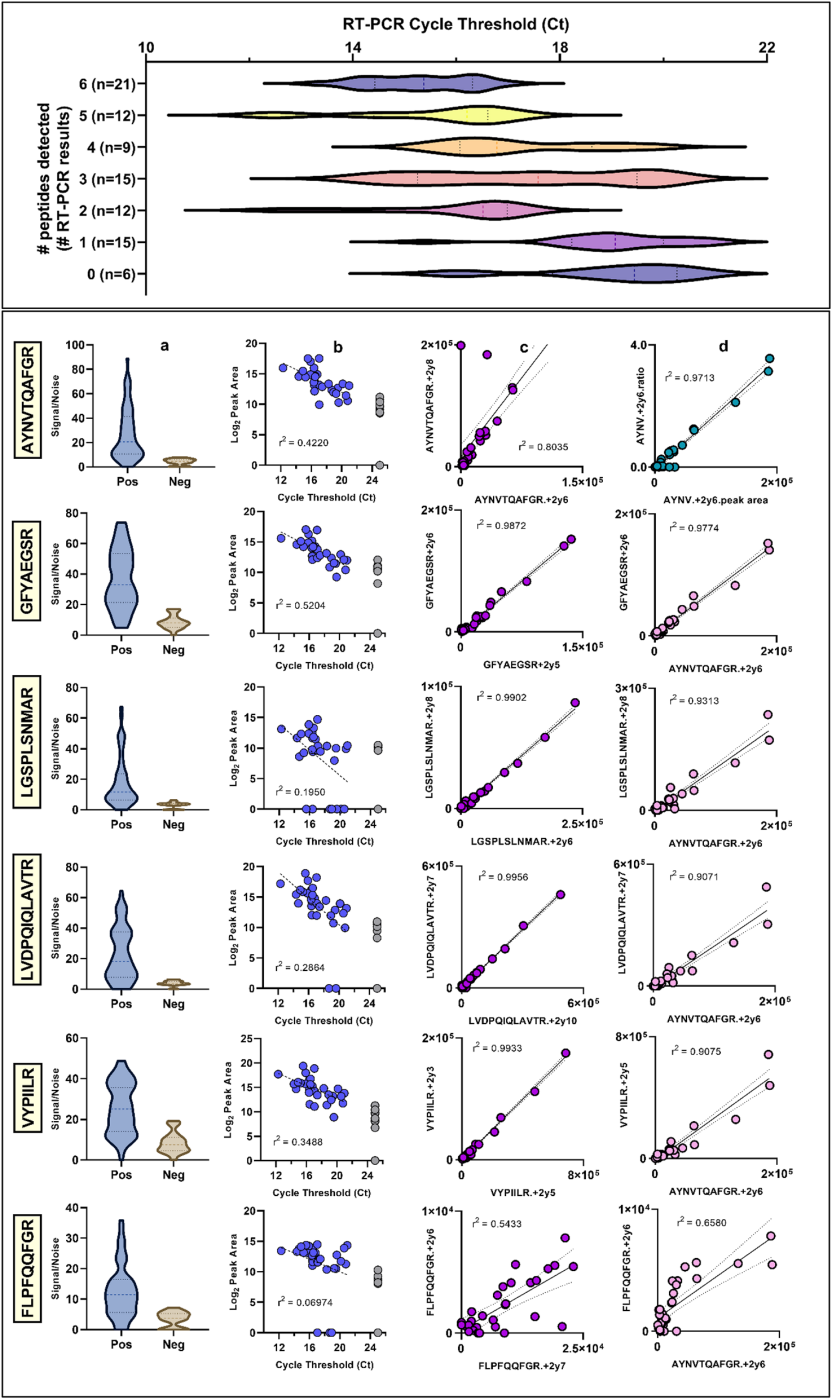
Targeted method performance for six peptides targeting 3 proteins from SARS-CoV-2. (Top panel) Spread of Ct values for 3 SARS-CoV-2 genes vs # peptides detected in clinical sample cohort 3. (Lower panel) From left; (**A**) Violin plot comparing software generated signal-to-noise ratio between positive (left) and negative (right) clinical specimens. (**B**) Correlation between reported N-gene Ct and peak area of the most intense MRM transition. Grey dots are peak areas detected in RT-PCR negative samples, assigned an arbitrary Ct of 25 for ease of reading. (**C**) linear regression of best two MRMs for each peptide for all RT-PCR positive samples. (**D**, top panel) linear regression and correlation for raw peak area and analyte/IS peak area ratio for peptide N.AYNTQAEFGR, which has been reported in other published targeted methods. (**D**, lower three panels) linear regression and correlation of other SARS-CoV-2 peptide peak areas to N.AYNTQAEFGR.

The final cohort of SARS-CoV-2 positive (n = 30) and negative (n = 10) samples was tested with the multi-virus LC–MS method, but with an increased digestion time. After an initial analysis showed detectable signals for the ORF9B peptide LVDPQIQLAVTR in 24 of the 30 RT-PCR positives, a confirmatory analysis including an additional MRM for that peptide as well as transitions for two other ORF9B peptides (VYPIILR and LGSPLSLNMAR) was performed. Considering the two most discriminating MRMs for each of 6 SARS-CoV-2 peptides and using an ion ratio tolerance of 30% and a retention time (RT) tolerance of ± 1% from the measured internal standard RT, 23 of 30 (77%) positive samples had detectable peaks for at least two peptides. There were no false positives from the 10 RT-PCR negative samples when the same criteria were applied. Reducing the threshold to 1 detected peptide increased the positive rate to 28/30 (93%) but increased the false positive rate to 20%. The best discriminating peptides were GFYAEGSR from the nucleoprotein and LVDPQIQLAVTR from the ORF9B protein (Fig. 4).

**Figure 5:**
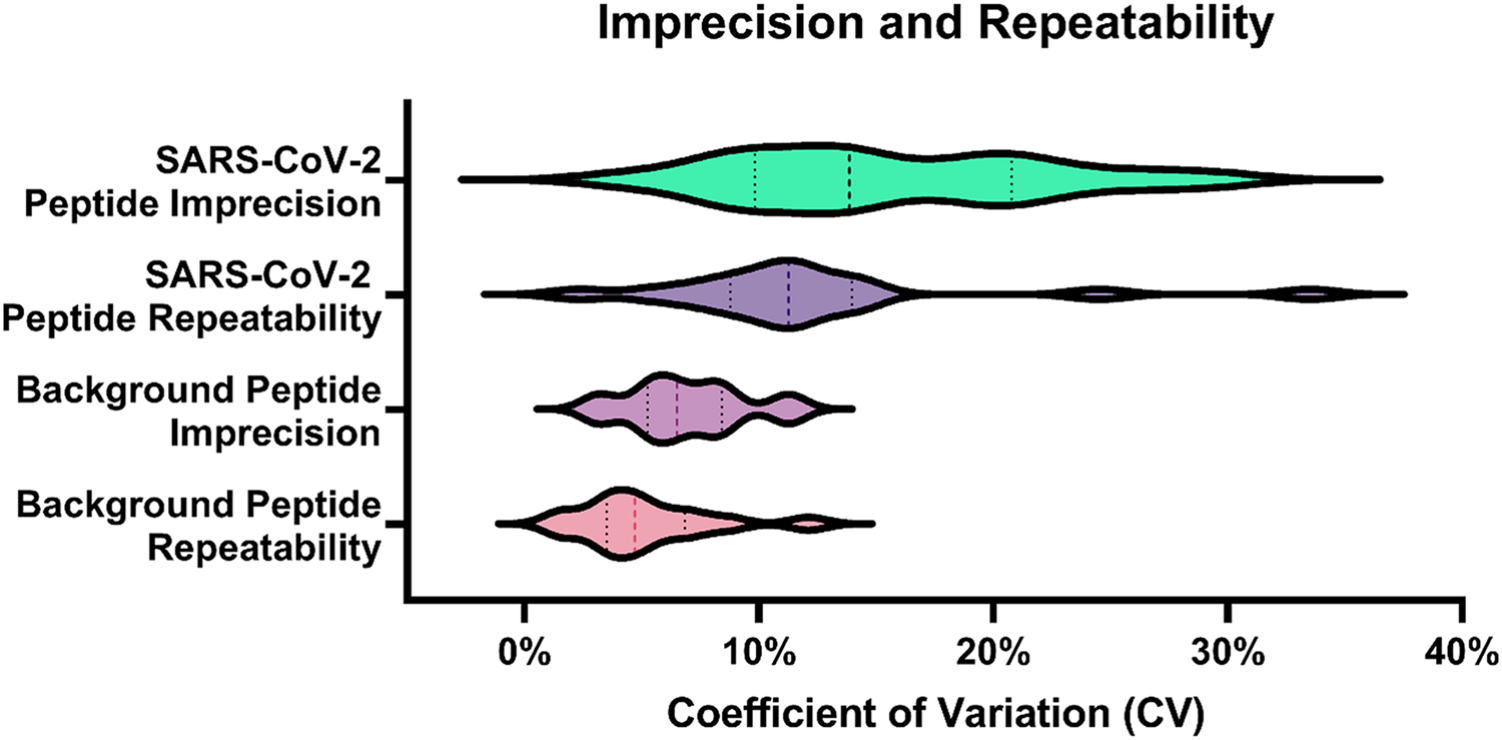
Targeted method imprecision (repeated preparations of pooled sample, *n* = 5) and repeatability (repeated injections of pooled sample extract, *n* = 6). Results are combined for 6 SARS-Cov-2 peptides using CVs for peak area, IS peak area and peak area ratio, and for 6 background peptides for peak area only.

Targeted method imprecision and robustness was briefly tested by analysis of 5 separate extractions of the same pooled sample, as well as replicate injections (n = 6) of a pooled extract. Method linearity was assessed across a small dynamic range by dilution of a pool of samples that showed high responses to SARS-CoV-2 proteins. Serial dilutions were made 1:1 with a pool of negative samples to mimic the log2 nature of Ct values. Unweighted linear least-squares regression showed the method was linear across the range surveyed (2^5^), with correlation coefficients ranging from 0.9669 to 0.9985 for IS-normalised peak areas vs relative concentration and from 0.97356 to 0.99992 for raw peak areas vs relative concentration (Supplementary table 6). Imprecision for peak areas, IS peak areas and IS area ratios ranged from 4.4% to 26.2% for SARS-CoV-2 peptides and 3.1% to 11.4% for background peptide peak areas. Repeatability ranged from 2.3% to 33.5% for peak areas, IS peak areas and IS area ratios of SARS-CoV-2 peptides and 1.6% to 12.1% for background peptide peak areas (Fig. 5 and Supplementary Table 7).

## Discussion

We utilised viral cultures to develop a targeted LC–MS/MS method as these contain acceptably high concentrations of viral proteins for testing of method parameters and require viral proteins to be liberated from virions for successful analysis. While it is possible to develop such method using recombinant viral proteins^30^, this approach omits the critical virion-extraction requirement for clinical specimens. Serum-free culture conditions removed the need for extensive sample fractionation and multiple LC–MS runs, yielding peptide identifications comparable to published studies where SDS-PAGE fractionation and 5 to 20 LC–MS runs were used in the analysis of FBS-supplemented viral cultures^27,29,31^. One notable omission in our protein list was the ORF 1ab polyprotein, detected with high sequence coverage in studies where infected host cells were harvested and digested^29,31^. In the search for clinical targets, however, viral proteins found in culture supernatants are more likely to be representative of those that can be easily sampled in the extracellular matrix of an infected patient, being either from secreted virions or viral proteins rather than from liberated endothelial cells. Further work is required to determine the relative contribution to the much-improved detection of virus proteins of; 1) smaller dynamic range of protein concentration being more compatible with LC–MS analysis, and 2) increased virus replication and secretion of virions occurring under “serum-free” conditions. However, these data suggest that serum-free cell culture conditions should be utilised where possible to rapidly acquire an in-depth snapshot of the viral proteome relevant to an infection.

Using viral culture supernatants to supply proteins for method development also revealed that the ORF9B protein held potential as a target in clinical samples, showing both high sequence coverage in discovery experiments and good sensitivity and specificity in targeted analysis of spiked nasopharyngeal swab extracts. ORF9B has so far been excluded from published targeted LC–MS methods, which have focused almost exclusively on the nucleoprotein, based on data suggesting that the latter exists in far greater abundance in the assessable virus proteome^31^. Although our initial analysis of targeted samples showed low method sensitivity overall, detecting SARS-CoV-2 proteins in only 2 of 10 RT-PCR positive samples, we noted that the ORF9B protein, via peptide LVDPQIQLAVTR, gave good responses in both positive detections and was thus retained for later analysis of a larger cohort of samples with lower RT-PCR Ct values. Confident detection of at least 1 peptide from ORF9B in approximately 80% of the cohort of low Ct SARS-CoV-2 RT-PCR positive samples is in agreement with reports of anti-ORF9B antibodies in serological analysis^32,33^ and suggests an under-explored role of this protein in SARS-CoV-2 infection.

Where previous works analysing clinical samples focused only on detection of SARS-CoV-2, the multiplexing capacity of MRM analysis was effectively demonstrated by our targeted method. For confident detection of the complete virus panel, a minimum of 16 proteotypic peptides (two peptides per virus, adapted from the accepted ‘two peptides per protein’ guideline for clinical protein MS^34^) were included, as well as transitions for isotope-labelled internal standard peptides and background proteins (Supplementary Table 1). Using short desalting and analytical columns ensured method washing and re-equilibration times were minimised. The relatively high flow rate applied to the core–shell analytical column ensured target peptides were optimally resolved as narrow peaks spread across the elution time. The final method required less than 1.5 min of MS acquisition time with minimal MRM concurrency and a total run time of ~ 3.25 min (Fig. 2). Combined with the rapid sample preparation procedure and simple data processing (compared to traditional proteomics database searching^35^), this method allows for the full analysis of a 96-well plate of samples in approximately 8 h. Linearity, imprecision, and repeatability under these conditions appear acceptable based on performance of the higher performing SARS-CoV-2 peptides and the background peptides, however further work is required to assess performance of the whole virus panel.

Monitoring peptides from human proteins allowed for assessment of differences in sampling of the clinical specimens. Peptides from human background proteins were selected from those known to be expressed abundantly in the nasopharyngeal space^36,37^ after analysis of a pool of SARS-CoV-2 negative nasopharyngeal swabs (n = 20). Inappropriate swab sampling has been confirmed to cause false negative RT-PCR results^38^. The addition of multiple background proteins to our targeted multi-virus detection method provided protection against falsely low results caused by sub-optimal sampling without the need for a secondary technique or extra reagents, an advantage that warrants further investigation.

The SARS-CoV-2 has been successfully cultured from clinical specimens with Ct values as high as 33^39^, indicating viral proteins may be present in samples with Ct values beyond those which we were able to designate as infected. However, the probability of successful culture is also less than 100% even with low Ct values^40^, suggesting a basis for the relatively poor correlations between Ct and viral protein concentration in the analysis reported here. Our detection limit corresponding with a Ct value of approximately 20 is consistent with other groups who assayed clinical samples using similar equipment and techniques and without virus protein enrichment^18,22^ when considered in light of the inter-assay variation amongst RT-PCR tests^41^. While we have not yet assessed the sensitivity of our method in terms of absolute protein concentration, we believe it would be comparable to other methods where similar samples, extraction techniques and instrumentation were used. In the absence of more sensitive and selective LC–MS instrumentation, immunocapture of viral proteins^19^ or peptides^21^ prior to LC–MS analysis has been shown to improve detection limits. However, while this may be possible for a single virus or small number of viruses, it may be prohibitively expensive for a virus panel applied to routine diagnostics. Strategies for depletion of background proteins may allow for higher volume of patient sample, and thus viral proteins, to be injected into the LC–MS. However, as a first step, nasopharyngeal swabs intended for MS analysis should not be extracted into VTM or other, similar protein rich solutions that limit sample loading and increase chromatographic interferences from isobaric species.

## Conclusion

We have presented an updated method for MS-based multi-virus testing proposed by others^13^. The sample throughput of our method improves on traditional proteomics approaches and compares well to the turnaround time of RT-PCR, while being accessible to many routine clinical LC–MS laboratories without the need for investment in new equipment. While the selectivity of our method is high, it is limited by the current sensitivity as a tool to be applied in the case of an inconclusive RT-PCR result for respiratory virus infection. However, our multiplexed test for the presence of eight respiratory viruses could potentially accommodate additional viruses and be quickly altered to track relevant viral mutations, indicating that LC–MS/MS may have future utility as a tool for differential diagnostics in suspected viral infection. As such, considering the importance of rapid and accurate diagnosis of viral infection to the current and future pandemics, efforts to incorporate the use of LC–MS/MS in clinical virology should continue.

## Materials and methods

### Clinical samples

Respiratory tract samples from nasopharyngeal swabs were collected in viral transport medium (VTM). All samples were initially stored at 4˚C, and those identified as positive for SARS-CoV-2 by RT-PCR were stored at -80 °C within 48 h of collection. Samples negative for SARS-CoV-2 were stored at room temperature in sample cohorts 1 and 2, and at -80 °C within 48 h of collection for cohort 3. Samples positive to other respiratory viruses were stored at 4 °C. This study was approved by the South Eastern Sydney Local Health District HREC (reference number 2021/ETH00108) and the NSW Health Pathology Research Governance Office (2021/STE01365). Experiments were conducted in accordance with the *National Statement on Ethical Conduct in Human Research (2007)* [https://www.nhmrc.gov.au/about-us/publications/national-statement-ethical-conduct-human-research-2007-updated-2018#toc__725 ]. Due to the use of deidentified specimens collected as part of standard diagnostic protocols, informed consent from all patients was waived.

Samples were tested for presence of SARS-CoV-2 RNA using standard commercial assays, predominantly with the Seegene Allplex platform for SARS-CoV-2^42^ and other respiratory viruses^43^. Any samples positive for SARS-CoV-2 RNA were further tested using the Roche Cobas 6800 platform (Roche USA) to confirm the presence of SARS-CoV-2 RNA and infer presence of SARS-CoV-2 virus.

### Viral cell culture

For viral culture, appropriate cell lines (Supplementary Table 1) were seeded in tissue culture plates 24 h prior. At the time of infection, tissue culture medium was removed, sub-confluent cellular monolayers were washed with PBS, and then inoculated with viral isolate. Plates were incubated for one hour before the inoculum was removed and replaced with viral culture media. Viral cultures were incubated at 37 °C (5% CO_2_) for 3–5 days and checked daily for cytopathic effect. Culture supernatants were collected, centrifuged at 2000rcf for 10 min to remove cellular debris, and stored at -80 °C as single-use aliquots.

### Protein extraction from viral cell culture

Virus-containing cell culture supernatant (150 µL) was mixed with methanol, chloroform, and water. All solvents were ice-cold at the time of addition. Centrifugation separated the phases, and the upper phase was removed and discarded. Methanol was added and the solution was vortexed for 30 s then centrifuged. The protein pellet was washed with methanol and allowed to air-dry before storage at −80 °C.

### Clinical specimen sample preparation

Proteins from respiratory tract samples (100 µL) were precipitated by mixing with sodium deoxycholate (SDC) and acetone^44^. After centrifugation, the pellet was washed with acetone, re-centrifuged and the protein pellet was air-dried.

### Protein preparation: standard conditions

Protein pellets were reconstituted in SDC (1% *w/w*), *n*-propanol (5% *v/v*), and triethylammonium bicarbonate (100 mM) and heated at 95 °C for 15 min at 600 rpm. Cysteine residues were reduced with dithiothreitol (DTT) and alkylated with iodoacetamide by incubating in the dark. The alkylation was quenched with DTT and the sample digested with trypsin (5 µg) overnight at 37 °C. Addition of formic acid (1:50 *v/v*) precipitated the SDC, which was removed by centrifugation. The supernatant was desalted using either Oasis MCX or HLB cartridges (Waters, Milford MA) according to the manufacturer’s protocol. Samples were dried and re-suspended in water with 3% acetonitrile and 0.1% formic acid (100 µL) for LC–MS/MS analysis.

### Protein preparation: rapid protocol

Protein pellets were reconstituted in Rapid Digest Buffer (100 µL, Sigma-Aldrich, St. Louis, MO) and sonicated for 5 min before the addition of SOLu-trypsin (5 µg, Sigma-Aldrich). Digestion was performed for 1 h (cohorts 1 and 2) or 2 h (cohort 3) at 60 °C, quenched with formic acid and 80 µL transferred to a 96-well plate for LC–MS/MS.

### High pH peptide fractionation

Peptides (approximately 15 µg) were fractionated using an Agilent 1290 UHPLC system (Agilent Technologies, Santa Clara CA) with an in-house packed capillary column. LC mobile phases were: (A) ammonium formate (10 mM, pH 7.9) and (B) water (10% *v/v*) and acetonitrile (90% *v/v*). Fractions were monitored by total UV absorbance.

### Nanoflow high performance liquid chromatography

Fractionated and unfractionated peptides were separated on self-packed fused-silica columns with incorporated emitter tips. Mobile phases were water (A) and acetonitrile (B) both with 0.1% formic acid. For Orbitrap analysis of fractionated peptide mixtures, a Thermo Scientific Ultimate 3000 LC (Thermo Fisher Scientific, Waltham MA) system operating in direct-injection mode was used at a flow rate of 400 nL/min with a 60 min gradient. For experiments using the TripleTOF 6600 (SCIEX, Framingham MA), the LC system was an Eksigent NanoLC 425 operating in direct-injection mode at a flow rate of 500 nL/min over a 75 min gradient.

### High-resolution mass spectrometry

Fractionated samples were analysed using a Q-Exactive Plus quadrupole-orbitrap mass spectrometer (Thermo Fisher Scientific) equipped with a nanoflow ion source operating in positive electrospray mode. The Orbitrap was operated in data-dependent acquisition mode. Unfractionated samples were analysed on a TripleTOF 6600 Quadrupole-Time-of-Flight (QTOF) mass spectrometer equipped with a NanoSpray III source (SCIEX). Untargeted analyses were performed in Information Dependent Acquisition (IDA) mode. A Data Independent Acquisition method (Sequential Windowed Acquisition of All Theoretical fragments, SWATH) for the TripleTOF 6600 was created using PeakView 2.2 and the SWATH Variable Window Calculator v1.0 (SCIEX) as described^45^.

### Discovery data processing

Orbitrap data were analysed using Proteome Discoverer v2.4 (Thermo) and Mascot v2.7 (Matrix Science, London) and TripleTOF IDA data was analysed using ProteinPilot software (v5.0.3, SCIEX, https://sciex.com/products/software/proteinpilot-software). Results were filtered to a global false discovery rate (FDR) of 1%. To facilitate cross-platform comparisons, PRIDE data sets were downloaded as .mgf (where available) or as .raw, processed with Proteome Discoverer v2.4 and exported as .mgf, then searched with ProteinPilot with settings appropriate for instruments used in each study.

Skyline (20.2.0.343, University of Washington) was used to extract chromatographic profiles and peak areas for virus peptides from SWATH raw data files for peptide selection.

### Peptide selection

Peptide sequences identified as unique in ‘no-species’ searches against the full Uniprot database with respiratory virus sequences appended were selected from DDA results as potential targets after confirmatory BLASTP searches. The filtered peptide list was assessed for peak shape and raw signal in SWATH data. Four MRM transitions per peptide were selected based on intensity and Q3 m/z being greater than Q1 m/z. Initial collision energy (CE) was calculated from precursor m/z and the rolling CE equations in Analyst TF 1.8 then optimised per MRM by repeated injections of cultures with CE set to 25%, 50%, 100%, 125% and 150% of the initial value. Peak areas at each CE were plotted in SCIEX OS Analytics (v1.7, SCIEX) and the optimum CE extrapolated from CE values generating the three highest responses.

### Targeted LC–MS/MS

Targeted multiple reaction monitoring (MRM) data acquisition was performed on a SCIEX QTRAP 6500^+^ coupled to an ExionLC AD UHPLC (SCIEX). The QTRAP 6500^+^ was operated in MRM mode with positive electrospray ionisation. The advanced scheduled MRM function of Analyst 1.7 (SCIEX) was used to schedule 62 MRMs across the acquisition time of 0.6 to 1.8 min after sample injection. The total sample run-time was completed in 3 min and 16 s.

### Targeted data processing

MRM data was processed using SCIEX OS 1.7 (SCIEX) with the MQ4 algorithm. Peak integration parameters were set to be appropriate for the peak shape and background noise found in the chromatogram from each MRM transition. Additional processing was performed using Microsoft Excel and GraphPad Prism v9.

## Supplementary Information

Supplementary Information.

## Data Availability

Mass spectrometry data used for viral protein and peptide identifications in serum-free culture have been deposited to the ProteomeXchange Consortium via the PRIDE^46^ partner repository with the dataset identifier PXD028562 and 10.6019/PXD028562.

## References

[1] Hasell J (2020). A cross-country database of COVID-19 testing. Sci. Data.

[2] Meyerowitz EA, Richterman A, Bogoch II, Low N, Cevik M (2021). Towards an accurate and systematic characterisation of persistently asymptomatic infection with SARS-CoV-2. Lancet. Infect. Dis.

[3] Wölfel R (2020). Virological assessment of hospitalized patients with COVID-2019. Nature.

[4] Bullard J (2020). Predicting infectious SARS-CoV-2 from diagnostic samples. Clin. Infect. Dis..

[5] Scohy A (2020). Low performance of rapid antigen detection test as frontline testing for COVID-19 diagnosis. J. Clin. Virol..

[6] Muhi S (2021). Multi-site assessment of rapid, point-of-care antigen testing for the diagnosis of SARS-CoV-2 infection in a low-prevalence setting: A validation and implementation study. Lancet Reg. Health Western Pacific.

[7] Scheiblauer H (2021). Comparative sensitivity evaluation for 122 CE-marked rapid diagnostic tests for SARS-CoV-2 antigen, Germany, September 2020 to April 2021. Eurosurveillance.

[8] Hoofnagle AN, Wener MH (2009). The fundamental flaws of immunoassays and potential solutions using tandem mass spectrometry. J. Immunol. Methods.

[9] Greco TM, Diner BA, Cristea IM (2014). The impact of mass spectrometry-based proteomics on fundamental discoveries in virology. Annu. Rev. Virol..

[10] Adaway JE, Keevil BG, Owen LJ (2015). Liquid chromatography tandem mass spectrometry in the clinical laboratory. Ann Clin Biochem.

[11] Cherkaoui A (2010). Comparison of two matrix-assisted laser desorption ionization-time of flight mass spectrometry methods with conventional phenotypic identification for routine identification of bacteria to the species level. J. Clin. Microbiol..

[12] Foster MW (2015). Targeted proteomics of human metapneumovirus in clinical samples and viral cultures. Anal. Chem..

[13] Majchrzykiewicz-Koehorst JA (2015). Rapid and generic identification of influenza A and other respiratory viruses with mass spectrometry. J. Virol. Methods.

[14] Gouveia D (2020). Proteotyping SARS-CoV-2 virus from nasopharyngeal swabs: A proof-of-concept focused on a 3 min mass spectrometry window. J. Proteome Res..

[15] Nikolaev EN (2020). Mass-spectrometric detection of SARS-CoV-2 virus in scrapings of the epithelium of the nasopharynx of infected patients via nucleocapsid N protein. J. Proteome Res..

[16] Singh P (2020). A rapid and sensitive method to detect SARS-CoV-2 virus using targeted-mass spectrometry. J. Proteins Proteomics.

[17] Ihling C (2020). Mass spectrometric identification of SARS-CoV-2 proteins from gargle solution samples of COVID-19 patients. J. Proteome Res..

[18] Cardozo KHM (2020). Establishing a mass spectrometry-based system for rapid detection of SARS-CoV-2 in large clinical sample cohorts. Nat. Commun..

[19] Renuse S (2021). A mass spectrometry-based targeted assay for detection of SARS-CoV-2 antigen from clinical specimens. EBioMedicine.

[20] Hober A (2021). Rapid and sensitive detection of SARS-CoV-2 infection using quantitative peptide enrichment LC-MS analysis. medRxiv.

[21] Mangalaparthi KK (2021). A SISCAPA-based approach for detection of SARS-CoV-2 viral antigens from clinical samples. Clin. Proteomics.

[22] Van Puyvelde B (2021). Cov-MS: A community-based template assay for mass-spectrometry-based protein detection in SARS-CoV-2 patients. JACS Au.

[23] Nachtigall FM, Pereira A, Trofymchuk OS, Santos LS (2020). Detection of SARS-CoV-2 in nasal swabs using MALDI-MS. Nat. Biotechnol..

[24] Rybicka M, Miłosz E, Bielawski KP (2021). Superiority of MALDI-TOF mass spectrometry over real-time PCR for SARS-CoV-2 RNA detection. Viruses.

[25] SoRelle JA, Patel K, Filkins L, Park JY (2020). Mass spectrometry for COVID-19. Clin Chem..

[26] Veldhoen M, Simas JP (2021). Endemic SARS-CoV-2 will maintain post-pandemic immunity. Nat. Rev. Immunol..

[27] Gouveia D (2020). Shortlisting SARS-CoV-2 peptides for targeted studies from experimental data-dependent acquisition tandem mass spectrometry data. Proteomics.

[28] Grenga L (2020). Shotgun proteomics analysis of SARS-CoV-2-infected cells and how it can optimize whole viral particle antigen production for vaccines. Emerg. Microb. Infect..

[29] Davidson AD (2020). Characterisation of the transcriptome and proteome of SARS-CoV-2 reveals a cell passage induced in-frame deletion of the furin-like cleavage site from the spike glycoprotein. Genome Med..

[30] Cazares LH (2020). Development of a parallel reaction monitoring mass spectrometry assay for the detection of SARS-CoV-2 spike glycoprotein and nucleoprotein. Anal. Chem..

[31] Bezstarosti K (2021). Targeted proteomics as a tool to detect SARS-CoV-2 proteins in clinical specimens. PLoS ONE.

[32] Jiang H-W (2020). SARS-CoV-2 proteome microarray for global profiling of COVID-19 specific IgG and IgM responses. Nat. Commun..

[33] Li Y (2021). Antibody landscape against SARS-CoV-2 reveals significant differences between non-structural/accessory and structural proteins. Cell Rep..

[34] Smit NPM (2021). The time has come for quantitative protein mass spectrometry tests that target unmet clinical needs. J. Am. Soc. Mass Spectrom..

[35] Verheggen K (2020). Anatomy and evolution of database search engines—A central component of mass spectrometry based proteomic workflows. Mass Spectrom. Rev..

[36] Sande CJ (2018). Untargeted analysis of the airway proteomes of children with respiratory infections using mass spectrometry based proteomics. Sci. Rep..

[37] Tomazic PV, Darnhofer B, Birner-Gruenberger R (2020). Nasal mucus proteome and its involvement in allergic rhinitis. Expert Rev. Proteomics.

[38] Kinloch NN (2020). Suboptimal biological sampling as a probable cause of false-negative COVID-19 diagnostic test results. J. Infect. Dis..

[39] La Scola B (2020). Viral RNA load as determined by cell culture as a management tool for discharge of SARS-CoV-2 patients from infectious disease wards. Eur. J. Clin. Microbiol. Infect. Dis..

[40] Gniazdowski V (2020). Repeat COVID-19 molecular testing: Correlation of SARS-CoV-2 culture with molecular assays and cycle thresholds. Clin. Infect. Dis..

[41] Evans D (2021). The dangers of using Cq to quantify nucleic acid in biological samples: A lesson from COVID-19. Clin Chem..

[42] Farfour E (2020). The Allplex 2019-nCoV (Seegene) assay: which performances are for SARS-CoV-2 infection diagnosis?. Eur. J. Clin. Microbiol. Infect. Dis..

[43] Wabe N (2021). Cepheid Xpert® Flu/RSV and Seegene Allplex™ RP1 show high diagnostic agreement for the detection of influenza A/B and respiratory syncytial viruses in clinical practice. Influenza Other Respir. Viruses.

[44] Nickerson JL, Doucette AA (2020). Rapid and quantitative protein precipitation for proteome analysis by mass spectrometry. J. Proteome Res..

[45] Krisp, C. & Molloy, M. P. in *Serum/Plasma Proteomics: Methods and Protocols* (eds David W. Greening & Richard J. Simpson) 373–383 (Springer New York, 2017).

[46] Perez-Riverol Y (2018). The PRIDE database and related tools and resources in 2019: improving support for quantification data. Nucleic Acids Res..

