# Supplementary material for: Simultaneous monitoring of eight human respiratory viruses including SARS-CoV-2 using liquid chromatography-tandem mass spectrometry
Source: Sci Rep. 2022 Aug 4;12:13392. doi: 10.1038/s41598-022-16250-y (PMC9352774; doi:10.1038/s41598-022-16250-y)
Supplement: Supplementary file 1 — Supplementary Information. [file 41598_2022_16250_MOESM1_ESM.pdf]

Supplementary information for

"Detection of Human Respiratory Viruses Including SARS-CoV-2 using Liquid Chromatography-Tandem Mass Spectrometry"

Christopher Hodgkins,[1,2] Laura K. Buckton,[1] Gregory J. Walker,[3,4] Ben Crossett,[5] Stuart J. Cordwell,[2,5] Andrea R. Horvath,[1] and William D. Rawlinson*.[1,3,4,6]

[1] NSW Health Pathology, Randwick, New South Wales, 2031, Australia.
[2] School of Life and Environmental Sciences and Charles Perkins Centre, the University of Sydney, 2006, Australia.
[3] Virology Research Laboratory, SAViD, Prince of Wales Hospital, Randwick, New South Wales, 2031, Australia.
[4] Schools of Medical Sciences, Women's and Children's Health, Faculty of Medicine and BABS Faculty of Science, University of New South Wales, Sydney, New South Wales, 2052, Australia.
[5] Sydney Mass Spectrometry, The University of Sydney, Sydney, New South Wales, 2006, Australia.
[6] Serology and Virology Division (SAViD), NSW Health Pathology, Randwick, New South Wales, 2031, Australia.

Table of Contents

Materials and methods 3
Viral cell culture 3
Table S1. Cell lines and media used for viral culture. 3
Protein extraction from viral cell culture 3
Clinical specimen sample preparation 3
Protein preparation: standard conditions 3
Protein preparation: rapid protocol 4
High pH Reversed-Phase LC Peptide Fractionation 4
Nanoflow and Microflow High Performance Liquid Chromatography 4
High-Resolution Mass Spectrometry 5
Targeted LC-MS/MS 6
Data processing 7
Results 8
Table S2. SARS-CoV-2 sequence coverage comparison. 8
Figure S1: Flow-rate-dependent sensitivity 9
Table S3. MRM table for multivirus panel. 10
Table S5: Detection of virus proteins in RT-PCR swab extracts 13
Table S6: Linearity of SARS-CoV-2 Peptide Response – raw- and IS-normalised response 14
Table S7: Peak Area results and calculated CVs for imprecision and repeatability experiments on pooled samples and sample extracts 15
Figure S2. Background Protein Monitoring: 17

Materials and methods

Viral cell culture

Table S1. Cell lines and media used for viral culture.

Virus	Cell line	Culture media
SARS-CoV-2	Vero E6	MEM + 2% FBS + 1x PSG
HCoV-229E	Vero E6	MEM + 2% FBS + 1x PSG
HCoV-OC43	MRC-5	MEM + 2% FBS + 1x PSG
IFV-A (H1N1pdm09)	MDCK	MEM + TPCK-treated trypsin (0.5 µg/mL) + 1x PSG
IFV-A (H3N2)	MDCK	MEM + TPCK-treated trypsin (0.5 µg/mL) + 1x PSG
IFV-B	MDCK	MEM + TPCK-treated trypsin (0.5 µg/mL) + 1x PSG
RSV-A	Vero	MEM + 2% FBS + 1x PSG
RSV-B	Vero	MEM + 2% FBS + 1x PSG

Protein extraction from viral cell culture

A 150 µL aliquot of virus-containing cell culture supernatant containing was mixed with 600 µL of ice-cold methanol, followed by 600 µL of ice-cold chloroform, then 450 µL cold water with vortex mixing for 30 seconds after each addition. The resulting mixture was centrifuged at 9,000 x *g* for 1 minute and the upper phase removed and discarded. 600 µL ice-cold methanol was added and the mixture was vortexed for 30 seconds then centrifuged at 9,000 x *g* for 2 minutes. The supernatant was removed and discarded, and the protein pellet washed with 450ul ice-cold MeOH, vortexed, and centrifuged at 9,000 x *g* for 1 minute. The supernatant was again removed, and the pellet allowed to air-dry before storage at -80°C.

Clinical specimen sample preparation

Protein from 100µL of respiratory tract sample was precipitated by addition of 20 µL sodium deoxycholate (final concentration 25 mM) and 400 µL acetone, followed by brief vortexing and centrifugation at 13,000 x *g* for 5 minutes.[18] The supernatant was removed, and the pellet washed with 400 µL acetone and centrifuged as described. The supernatant removed and discarded, and the protein pellet was air-dried for 20 minutes at room temperature.

Protein preparation: standard conditions

Protein pellets were reconstituted in 1% sodium deoxycholate (SDC), 5% *n*-propanol and 100 mM triethyl ammonium bicarbonate (TEAB) and heated at 95°C for 15 minutes at 600 rpm. For reduction and alkylation of cysteine residues, 10 µL dithiothreitol (DTT, final concentration 10 mM) was added and the sample heated to 60°C for 50 minutes, after which it was cooled and mixed with 10 µL iodoacetamide (final concentration 20 mM) and incubated in the dark for 30 minutes. Alkylation was quenched with 2 µL DTT (final concentration 25 mM) and incubated in the dark for 15 minutes. The sample was digested with 5 µg of trypsin overnight at 37°C. Addition of 1:50 *v/v* formic acid precipitated the SDC, which was pelleted by centrifugation. The supernatant was desalted using either Oasis MCX or HLB 10mg cartridges according to the manufacturer's protocol. Samples were evaporated to dryness and re-suspended in 100 µL 0.1% formic acid in 97:3 water:acetonitrile (*v/v*) for LC-MS/MS analysis.

Protein preparation: rapid protocol

Proteins pellets were reconstituted in 100 µL Rapid Digest Buffer (Sigma-Aldrich, St. Louis, MO) and sonicated for 5 minutes before the addition of 5 µg of SOLu-trypsin (Sigma). Digestion was performed for 1 hour at 60°C, quenched with 1:50 *v/v* formic acid, and transferred to a 96-well plate for LC-MS/MS analysis.

High pH Reversed-Phase LC Peptide Fractionation

Approximately 15 µg peptides were loaded on to an in-house packed 320 µm × 25 cm column (3.5 µm particle size, Xbridge BEH C18; Waters, Milford, MA, USA). LC mobile phase buffers were comprised of A: 10 mM ammonium formate, pH 7.9 and B: 9:1 acetonitrile:water (*v/v*) water. Peptides were eluted using a linear gradient of 5% to 50% B over 45 mins at a flow rate of 6 µL/min. Twelve concatenated fractions were then dried down prior to LC-MS analysis.

Nanoflow and Microflow High Performance Liquid Chromatography

Tryptic digests were separated on a self-packed fused-silica column 100 µm x 30 cm (1.9 µm particle size, GmbH Germany) and pulled to an emitter tip. Mobile phases were water and acetonitrile both with 0.1% formic acid. For Orbitrap analysis of fractionated peptide mixtures, a Thermo Scientific Ultimate 3000 LC system operating in direct-injection mode was used at a flow rate of 400 µL/min

with a 60-minute gradient from 5% to 40% B. For nanoflow experiments using the TripleTOF 6600, the LC system was an Eksigent NanoLC 425 operating in direct-injection mode at a flow rate of 500 µL/min over a 75-minute gradient. For microflow experiments using the TripleTOF 6600, the LC system was an Eksigent NanoLC 415 equipped with a 5-50µL flow cell. Peptide separations were performed over a 30-minute gradient on a Kinetex C18 0.3 x 150mm, 2.6µm column (Phenomenex, Torrance CA) at 7.5µL/min.

High-Resolution Mass Spectrometry

Fractionated samples were analysed using a Q-Exactive Plus quadrupole-orbitrap mass spectrometer (Thermo Fisher Scientific, Waltham MA) equipped with a nanoflow ion source operating in positive electrospray mode. Column voltage was 2300 V and the heated capillary set to 275°C. The Orbitrap was operated in data-dependent acquisition mode. A survey scan of 350-1550 *m/z* was acquired (resolution = 70,000, with an accumulation target value of 1,000,000 ions). Up to 10 of the most abundant ions (>1.7e5 ions), with charge states ≥ +3 were sequentially isolated and fragmented and target value of 100,000 ions collected. Ions selected for MS/MS were dynamically excluded for 20 s. The data were analysed using Proteome Discoverer v2.4 (Thermo) and Mascot v2.7 (Matrix Science, London). The search parameters included the following variable modifications: carbamylation (C), oxidized methionine, protein N-terminal acetylation and deamination (NQ), and trypsin specified as the enzyme and 10 ppm precursor mass tolerance. The search databases included common contaminants, human proteome (Uniprot) and SARS-CoV-2 proteome. Unfractionated samples were analysed on a TripleTOF 6600 Quadrupole-Time-of-Flight (QTOF) mass spectrometer equipped with a NanoSpray III source for nanoflow experiments and a DuoSpray source with a 50µm electrode for microflow experiments. Source Parameters were ISVF = 3200, GS1 = 25, CUR = 35, Interface Heater Temperature (IHT) = 175°C and CUR = 35, GS1 = 20, GS2 = 30, ISVF = 5500, TEM = 150°C for NanoSpray III and DuoSPray experiments, respectively. Untargeted analysis was performed in Information Dependent Acquisition (IDA) mode, where in each cycle a TOF survey scan from *m/z* 350 – 1,500 was accumulated for 250 milliseconds (ms), followed by up to 80 MS/MS spectra collected using dynamic accumulation with a minimum time of 25 ms per precursor. MS/MS collection was triggered by precursors with intensity greater than 1000 cps,

charge state from 2+ to 5+, excluding isotopes within 4 Da, mass tolerance = 20 ppm. Selected precursors were excluded from collection for 60 seconds after 2 occurrences.

A Data Independent Acquisition method (Sequential Windowed Acquisition of All Theoretical fragments, or SWATH) for the TripleTOF 6600 was created using PeakView 2.2 and the SWATH Variable Window Calculator v1.0 (SCIEX). In each cycle, a TOF-MS scan from *m/z* 350 – 1,500 was first collected for 50 ms, followed by 100 variably sized SWATH windows where precursors in each window are isolated in Q1, co-fragmented in the collision cell and fragments from 350 - 1,500 *m/z* are separated and recorded by the time-of-flight mass analyser. Each SWATH window was accumulated for 25 ms using collision energy calculated for 2+ charges with the rolling CE equations of Analyst TF v1.7 (SCIEX) and a collision energy spread of 3 V.

Targeted LC-MS/MS

Targeted MRM data acquisition was performed on a QTRAP 6500^{+} coupled to an ExionLC AD system equipped with four ExionLC AD pumps, a Multiplate Sampler and AC column oven fitted with two column switching valves (SCIEX, Framingham MA). An additional, external 6-port/2-position valve (Shimadzu) was controlled via contact closure from the LC system. A Phenomenex Gemini C18 column (2 mm x 20 mm, 3 µm) was used for online desalting, before being switched in-line with a Cortecs C18 2.1 mm x 50 mm, 2.7 µm column (Waters, Milford MA). Mobile phases were 0.1% formic acid in water (A) and acetonitrile (B) for both sets of pumps. 10 µL of sample was loaded at a flow rate of 1,600 µL/min onto the trapping column for 0.1 minutes, at which time the trap and analytical columns were connected by the switching valve and a non-linear gradient applied to elute peptides from the sample. At 1.8 minutes, the valve switched the trap column off-line with the analytical column so the most hydrophobic sample components could be washed directly to waste by the loading pumps.

The QTRAP 6500+ was operated in multiple reaction monitoring (MRM) mode with positive electrospray ionisation. Flow from the LC was split at a 1:1 ratio by means of a PEEK t-piece placed immediately before the source. Source parameters were IS = 5500, GS1 = 50, GS2 = 55, CUR = 35. The advanced scheduled MRM function of Analyst 1.7 (SCIEX) was used to schedule 62 MRMs

across the acquisition time, which was set by using scheduled ionisation from 0.6 to 1.8 minutes after sample injection. The total target cycle time was 0.12 seconds, with pause times set to 2 ms and MRM acquisition windows 8 seconds for virus peptides and 6 seconds for background peptides. The total injection cycle including loading, desalting, peptide elution, washing and re-equilibration for each sample was completed in 3 minutes and 16 seconds.

Data processing

Sequence matching of TripleTOF IDA data was performed using ProteinPilot software (v5.0.3, SCIEX, https://sciex.com/products/software/proteinpilot-software). A custom FASTA file was constructed by concatenating FASTA files for Homo sapiens, Chlorocebus sabaeus, Bos taurus, SARS-CoV-2, 229E, OC43, Influenza A (H1N1 and H3N2) and Influenza B, RSV (A and B), downloaded from uniprot.org, along with a list of common contaminating proteins supplied with ProteinPilot software. Results were filtered to a global false discovery rate of 1%.

Skyline (20.2.0.343, University of Washington) was used to extract chromatographic profiles and peak areas for virus peptides from SWATH raw data files. Peptides were selected as matching to a library extracted from the ProteinPilot result file for the serum-free culture sample, with uniqueness enforced at the protein level in a background proteome generated from the multi-organism FASTA file described above. +2 or +3 precursor charge states were allowed, along with y or b ions selected for picked intensity in the library. Four fragment ions per precursor were used for peak area extraction.

MRM data was processed using SCIEX OS 1.7 (SCIEX) with the MQ4 algorithm. Peak integration parameters were set to be appropriate for the peak shape and background noise found in the chromatogram from each MRM transition.

Results

Table S2. SARS-CoV-2 sequence coverage comparison.

Comparison of SARS-CoV-2 protein sequence coverage at high confidence (>95%) for this study in standard (2% FBS) and serum-free culturing conditions versus public datasets reprocessed through the same pipeline and matched to the same sequence database. Length of blue bars represents % sequence coverage while overlaid numbers are number of unique peptide sequences identified. Note: in the event of a missed cleavage during tryptic digestion, there will be overlap between sequences identified as unique, resulting in an imperfect correlation between number of peptides and sequence coverage.

	Envelope	Membrane	Nucleoprotein	Spike Glycoprotein	ORF3a	ORF6	ORF7a	ORF8	ORF9B	Replicase polyprotein 1ab
**2% FBS Culture**		1	14	6				1	5	
**Serum-free Culture**		9	39	23			1	4	13	
**Grenga et al.**		5	25	17	1					4
**Gouveia et al.**		8	46	31	2			1	5	15
**Davidson et al.**	11	4	59	66	3	1	4	3	11	244

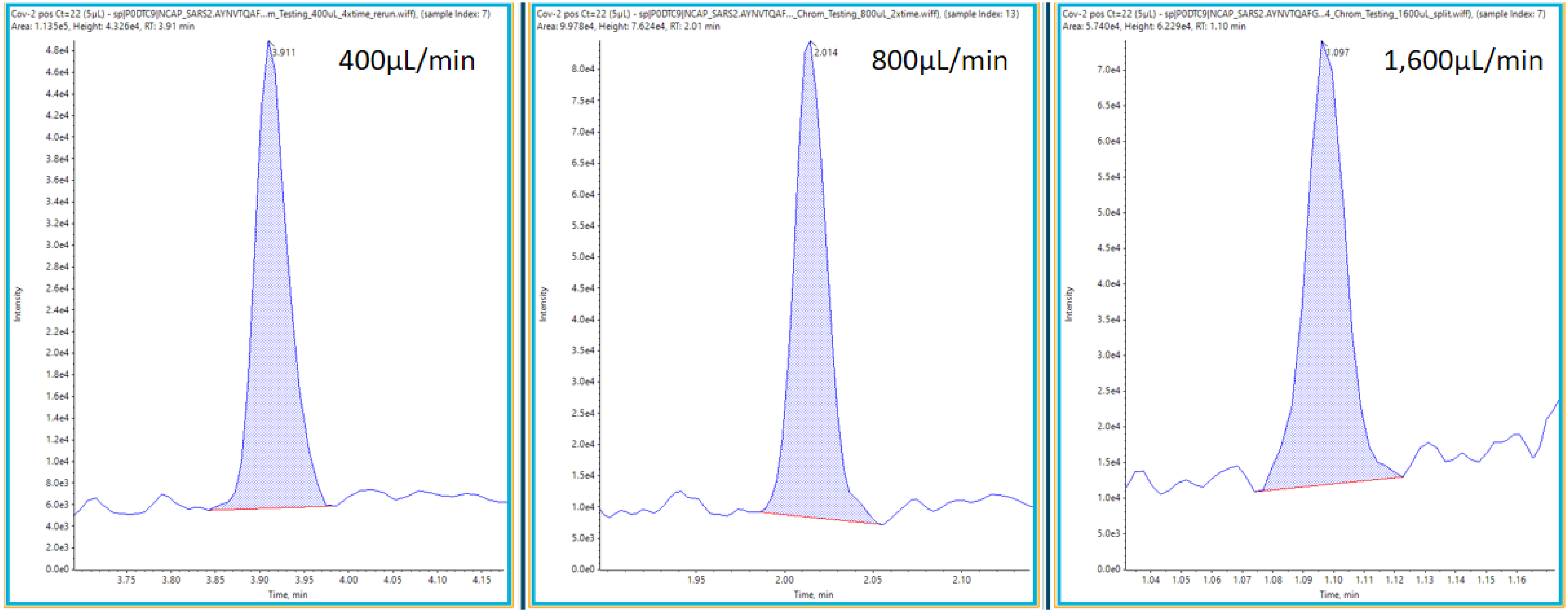

Figure S1: Flow-rate-dependent sensitivity

Comparison of peptide AYNVTQAFGR +2y6 from a SARS-CoV-2 positive sample at flow rates of 400, 800 and 1,600µL/min. Increasing flow to 1,600µL/min with 1:1 flow split allowed for run time to be halved vs 800µL/min with only a small reduction in signal-to-noise ratio for positive samples.

Table S3. MRM table for multivirus panel. Peptides with an isotope labelled surrogate are labelled with an *.

Organism	Protein	Peptide	Precursor charge state	Fragment ion	Q1	Q3	Retention time (min)	Collision energy
SARS-CoV-2	Nucleoprotein	AYNVTQAFGR	+2	y8	563.786	892.464	1.18	24
		AYNVTQAFGR	+2	y6	563.786	679.352	1.18	26
		GFYAEGSR	+2	y6	443.706	682.315	0.76	21
		GFYAEGSR	+2	y5	443.706	519.252	0.76	22
	ORF 9B	LVDPQIQLAVTR	+2	y7	676.898	800.499	1.47	40
		LVDPQIQLAVTR	+2	y10	676.898	1140.637	1.47	30
		LGSPLSLNMAR	+2	y6	579.819	691.356	1.65	33
		LGSPLSLNMAR	+2	y8	579.819	901.492	1.65	29
	Nucleoprotein	AYNVTQAFGR*	+2	y8	568.79	902.472	1.18	24
		AYNVTQAFGR*	+2	y6	568.79	689.36	1.18	26
		GFYAEGSR*	+2	y6	448.71	692.324	0.76	21
		GFYAEGSR*	+2	y5	448.71	529.26	0.76	22
	ORF 9B	LGSPLSLNMAR*	+2	y6	584.823	701.364	1.65	33
		LGSPLSLNMAR*	+2	y8	584.823	911.501	1.65	29
		LVDPQIQLAVTR*	+2	y7	681.903	810.507	1.47	40
		LVDPQIQLAVTR*	+2	y10	681.903	1150.645	1.47	30
Homo Sapiens	Lactotransferrin	FQLFGSPSGQK	+2	y9	598.309	920.484	1.4	28
		FQLFGSPSGQK	+2	y8	598.309	807.4	1.4	28
		LRPVAAEVYGTER	+3	y5	487.6	625.294	1	21
		LRPVAAEVYGTER	+3	b7	487.6	737.43	1	21
	Albumin	AEFAEVSK	+2	y5	440.724	533.293	0.8	21
		AEFAEVSK	+2	y6	440.724	680.361	0.8	21
		FQNALLVR	+2	y5	480.785	571.393	1.25	23
		FQNALLVR	+2	y6	480.785	685.436	1.25	23
		LVNEVTEFAK	+2	y6	575.311	694.377	1.23	27
		LVNEVTEFAK	+2	y8	575.311	937.463	1.23	27
	Myeloperoxidase	IANVFTNAFR	+2	y5	576.812	608.315	1.56	27

		IANVFTNAFR	+2	y6	576.812	755.383	1.56	27
	BPI Fold Containing Family B Member 1	ILTQDTPEFFIDQGHAK	+3	y7	653.999	768.4	1.53	29
		ILTQDTPEFFIDQGHAK	+3	y8	653.999	915.468	1.53	29
Bos taurus	Albumin	DAFLGSFLYEYSR	+2	y5	784.375	717.32	1.74	60
		DAFLGSFLYEYSR	+2	y10	784.375	1234.61	1.74	60
Human Coronavirus 229E	Nucleoprotein	WADASEPQR	+2	y7	530.246	802.369	0.72	25
		WADASEPQR	+2	y8	530.246	873.406	0.72	25
		VEGVVWVAVDGAK	+2	y8	664.864	845.452	1.54	32
		VEGVVWVAVDGAK	+2	y9	664.864	944.52	1.54	32
Human Coronavirus OC43	Nucleoprotein	ELTAEDISLLK	+2	y7	616.343	817.467	1.57	29
		ELTAEDISLLK	+2	y9	616.343	989.551	1.57	29
	Membrane Protein	GSGMDTALLR	+2	y6	510.761	688.399	1.17	24
		GSGMDTALLR	+2	y8	510.761	876.461	1.17	24
Human Respiratory Syncytial Virus A	Matrix Protein	FAIKPMED	+2	y4	475.736	491.181	1.19	22
		FAIKPMED	+2	y6	475.736	732.36	1.19	22
	Matrix M2-1	SITINNPK	+2	y6	443.75	686.4	0.79	19
		SITINNPK	+2	y5	443.75	585.3	0.79	19
Human Respiratory Syncytial Virus B	Matrix Protein	FSIKPLED	+2	y6	474.75	714.4	1.34	23
		FSIKPLED	+2	y7	474.75	801.4	1.34	23
	Matrix M2	SITISNPK	+2	y6	430.24	659.4	0.82	18
		SITISNPK	+2	y4	430.24	445.24	0.82	18
Human Respiratory Syncytial Virus A&B	Matrix Protein	VIIPTYLR	+2	y5	487.805	649.367	1.48	23
		VIIPTYLR	+2	y6	487.805	762.451	1.48	23
Influenza B	Nucleoprotein	TNPIEIPIK	+2	y7+2	512.805	405.26	1.38	24
		TNPIEIPIK	+2	y7	512.805	809.513	1.38	24
		GGGTLVAEAIR	+2	y5	522.296	559.32	1.17	25
		GGGTLVAEAIR	+2	y6	522.296	658.388	1.17	25
	Non-structural Protein 1	LVATDDLTVEDEEDGHR	+3	y8	638.629	986.381	1.09	35
		LVATDDLTVEDEEDGHR	+3	y10	638.629	1186.497	1.09	35
Influenza A (H1N1)	Nucleoprotein	ASAGQTSVQPTFSVQR	+2	y8	832.424	962.505	1.1	40
		ASAGQTSVQPTFSVQR	+2	y9	832.424	1061.574	1.1	40

Influenza A (H3N2)	Nucleoprotein	ASAGQISVQPTFSVQR	+2	y10	838.442	1148.606	1.29	40
		ASAGQISVQPTFSVQR	+3	y7	559.297	834.447	1.29	25
Influenza A (H1N1 & H3N2)	Nucleoprotein	NPGNAEIEDLIFLAR	+2	y9	836.439	1089.63	1.75	40
		NPGNAEIEDLIFLAR	+2	y10	836.439	1218.673	1.75	40

Table S5: Detection of virus proteins in RT-PCR swab extracts by LC-MS/MS – cohort 1 and 2

VTM swab number	Virus	PCR Ct	Detect by LC-MS?
1	SARS-Cov-2	36	No
2	SARS-Cov-2	34	No
3	SARS-Cov-2	37	No
4	SARS-Cov-2	33	No
5	SARS-Cov-2	32	No
6	SARS-Cov-2	20	No
7	SARS-Cov-2	17	Yes
8	SARS-Cov-2	22	No
9	SARS-Cov-2	25	No
10	SARS-Cov-2	19	Yes
11	RSV A	15	Yes
12	RSV A	17	Yes
13	RSV A	22	Yes
14	RSV A	24	No
15	OC43	29	No
16	OC43	30	No

Table S6: Linearity of SARS-CoV-2 Peptide Response – raw- and IS-normalised response

Peptide	R^2 (no IS)	R^2 (with IS)
sp|P0DTC9|NCAP_SARS2.AYNVTQAFGR.+2y6.light	0.99588	0.96686
sp|P0DTC9|NCAP_SARS2.AYNVTQAFGR.+2y8.light	0.99883	0.99584
sp|P0DTC9|NCAP_SARS2.GFYAEGSR.+2y5.light	0.99474	0.99757
sp|P0DTC9|NCAP_SARS2.GFYAEGSR.+2y6.light	0.99480	0.99803
sp|P0DTD2|ORF9B_SARS2.LGSPLSLNMAR.+2y10.light	0.99310	0.96172
sp|P0DTD2|ORF9B_SARS2.LGSPLSLNMAR.+2y6.light	0.98263	0.97788
sp|P0DTD2|ORF9B_SARS2.LGSPLSLNMAR.+2y7.light	0.97356	0.9648
sp|P0DTD2|ORF9B_SARS2.LGSPLSLNMAR.+2y8.light	0.98023	0.98033
sp|P0DTD2|ORF9B_SARS2.LVDPQIQLAVTR.+2y10.light	0.99992	0.99499
sp|P0DTD2|ORF9B_SARS2.LVDPQIQLAVTR.+2y6.light	0.99448	0.99278
sp|P0DTD2|ORF9B_SARS2.LVDPQIQLAVTR.+2y7.light	0.99545	0.96929
sp|P0DTD2|ORF9B_SARS2.LVDPQIQLAVTR.+2y9.light	0.99866	0.98528
sp|P0DTD2|ORF9B_SARS2.VYPIILR.+2y3.light	0.99890	0.98091
sp|P0DTD2|ORF9B_SARS2.VYPIILR.+2y5.light	0.99901	0.99723
MIN	0.97356	0.96172
MAX	0.99992	0.99803
AVERAGE	0.99287	0.98311

Table S7: Peak Area results and calculated CVs for imprecision and repeatability experiments on pooled samples and sample extracts

	Imprecision	Rep 1	Rep 2	Rep 3	Rep 4	Rep 5	%CV		Repeatability	Rep 1	Rep 2	Rep 3	Rep 4	Rep 5	Rep 6	%CV
N.GFYAEGSR.+2y6	Area	1.46E+04	1.40E+04	1.88E+04	1.57E+04	1.40E+04	13.0%		Area	1.39E+04	1.43E+04	1.42E+04	1.96E+04	1.77E+04	1.69E+04	14.4%
	IS Area	5.64E+04	4.01E+04	5.79E+04	5.71E+04	5.20E+04	14.1%		IS Area	4.47E+04	5.06E+04	4.81E+04	5.09E+04	5.00E+04	5.23E+04	5.4%
	Area Ratio	2.59E-01	3.50E-01	3.24E-01	2.75E-01	2.69E-01	13.4%		Area Ratio	3.11E-01	2.84E-01	2.95E-01	3.84E-01	3.53E-01	3.24E-01	11.6%
N.AYNVTQAFGR.+2y6	Area	1.84E+04	1.54E+04	1.96E+04	1.88E+04	1.88E+04	9.0%		Area	1.67E+04	1.46E+04	1.71E+04	1.65E+04	1.82E+04	1.66E+04	7.1%
	IS Area	9.63E+04	9.50E+04	9.47E+04	8.67E+04	8.96E+04	4.4%		IS Area	8.64E+04	8.12E+04	9.25E+04	7.74E+04	7.09E+04	8.69E+04	9.3%
	Area Ratio	1.91E-01	1.62E-01	2.07E-01	2.17E-01	2.10E-01	11.1%		Area Ratio	1.93E-01	1.80E-01	1.85E-01	2.13E-01	2.57E-01	1.91E-01	14.1%
ORF9B.LGSPLSNMAR.+2y6	Area	9.22E+03	9.54E+03	1.19E+04	9.39E+03	1.45E+04	21.0%		Area	1.20E+04	6.36E+03	6.46E+03	9.39E+03	9.74E+03	8.75E+03	24.5%
	IS Area	2.14E+04	2.29E+04	1.81E+04	2.19E+04	3.04E+04	19.8%		IS Area	1.41E+04	1.64E+04	1.83E+04	1.52E+04	1.96E+04	1.61E+04	12.2%
	Area Ratio	4.32E-01	4.17E-01	6.56E-01	4.29E-01	4.78E-01	20.7%		Area Ratio	8.55E-01	3.88E-01	3.53E-01	6.17E-01	4.96E-01	5.44E-01	33.5%
ORF9B.LVDPQIQLAVTR.+2y10	Area	1.76E+04	1.79E+04	2.42E+04	2.18E+04	2.49E+04	16.2%		Area	2.16E+04	1.48E+04	1.77E+04	1.56E+04	1.63E+04	1.78E+04	13.9%
	IS Area	7.09E+04	5.62E+04	6.90E+04	7.35E+04	7.16E+04	10.1%		IS Area	5.83E+04	4.84E+04	5.92E+04	5.33E+04	5.75E+04	5.08E+04	8.1%
	Area Ratio	2.48E-01	3.18E-01	3.52E-01	2.96E-01	3.47E-01	13.6%		Area Ratio	3.71E-01	3.07E-01	3.00E-01	2.93E-01	2.84E-01	3.49E-01	10.9%
ORF9B.VYPIILR.+2y5	Area	2.21E+04	3.10E+04	2.97E+04	3.46E+04	3.75E+04	18.8%		Area	2.70E+04	2.33E+04	2.43E+04	2.35E+04	1.98E+04	2.21E+04	10.2%
	IS Area	8.70E+04	6.86E+04	7.53E+04	7.77E+04	7.46E+04	8.7%		IS Area	7.63E+04	6.08E+04	6.05E+04	6.25E+04	6.73E+04	6.61E+04	9.0%
	Area Ratio	2.54E-01	4.52E-01	3.95E-01	4.45E-01	5.03E-01	23.2%		Area Ratio	3.54E-01	3.84E-01	4.01E-01	3.76E-01	2.93E-01	3.35E-01	10.9%
S.FLPFQQFGR+2y7	Area	9.59E+03	4.73E+03	7.34E+03	8.49E+03	9.94E+03	26.2%		Area	6.05E+03	8.31E+03	7.46E+03	7.70E+03	8.22E+03	6.85E+03	11.6%
	IS Area	2.50E+05	2.24E+05	2.38E+05	2.33E+05	2.02E+05	7.9%		IS Area	2.15E+05	2.19E+05	2.21E+05	2.14E+05	2.09E+05	2.09E+05	2.3%
	Area Ratio	3.83E-02	2.11E-02	3.08E-02	3.65E-02	4.93E-02	29.4%		Area Ratio	2.82E-02	3.80E-02	3.38E-02	3.60E-02	3.94E-02	3.27E-02	11.7%
Human.ALB.AEFAEVSK.+2y5	Area	2.74E+06	2.62E+06	2.74E+06	2.80E+06	2.59E+06	3.3%		Area	2.53E+06	2.50E+06	2.51E+06	2.38E+06	2.45E+06	2.62E+06	3.2%
Human.ALB.AEFAEVSK.+2y6	Area	1.07E+07	1.03E+07	1.06E+07	1.12E+07	1.06E+07	3.1%		Area	1.04E+07	1.01E+07	1.04E+07	9.60E+06	9.84E+06	1.06E+07	3.6%
Human.TRFL.LRPVAAVYGTER.+3b7	Area	2.93E+05	3.47E+05	3.45E+05	3.19E+05	3.30E+05	6.8%		Area	2.60E+05	2.98E+05	2.85E+05	2.66E+05	2.94E+05	2.93E+05	5.6%
Human.TRFL.LRPVAAVYGTER.+3y6	Area	2.91E+05	3.54E+05	3.50E+05	3.26E+05	3.30E+05	7.6%		Area	2.67E+05	2.90E+05	2.85E+05	2.78E+05	3.05E+05	3.02E+05	4.9%
Human.ALB.LVNEVTEFAK.+2y6	Area	2.63E+06	2.70E+06	3.14E+06	3.10E+06	3.07E+06	8.2%		Area	2.60E+06	2.61E+06	2.59E+06	2.55E+06	2.51E+06	2.60E+06	1.6%
Human.ALB.LVNEVTEFAK.+2y8	Area	1.03E+07	1.03E+07	1.17E+07	1.15E+07	1.15E+07	6.2%		Area	9.98E+06	9.84E+06	9.70E+06	9.73E+06	9.51E+06	9.66E+06	1.6%
Human.ALB.FQNALLVR.+2y5	Area	2.80E+06	2.91E+06	3.23E+06	3.20E+06	3.15E+06	6.2%		Area	2.83E+06	2.54E+06	2.54E+06	2.59E+06	2.53E+06	2.51E+06	4.7%
Human.ALB.FQNALLVR.+2y6	Area	1.50E+07	1.50E+07	1.65E+07	1.61E+07	1.70E+07	5.6%		Area	1.50E+07	1.42E+07	1.35E+07	1.39E+07	1.43E+07	1.38E+07	3.7%
Human.TRFL.FQLFGSPSGQK.+2y8	Area	1.78E+05	2.18E+05	2.34E+05	1.99E+05	1.85E+05	11.4%		Area	1.86E+05	1.64E+05	1.78E+05	1.73E+05	1.53E+05	1.71E+05	6.7%

Human.TRFL.FQLFGSPSGQK.+2y9	Area	2.09E+05	2.77E+05	2.75E+05	2.47E+05	2.43E+05	11.1%		Area	1.96E+05	2.08E+05	2.21E+05	1.99E+05	2.10E+05	2.17E+05	4.7%
Human.BPIB1.ILTQDTPEFFIDQGHAK.+3y7	Area	3.02E+05	3.07E+05	3.45E+05	3.20E+05	3.15E+05	5.3%		Area	2.81E+05	2.52E+05	2.66E+05	2.55E+05	2.60E+05	2.61E+05	3.8%
Human.BPIB1.ILTQDTPEFFIDQGHAK.+3y8	Area	2.17E+05	2.07E+05	2.47E+05	2.49E+05	2.13E+05	8.7%		Area	1.92E+05	1.76E+05	1.79E+05	1.80E+05	2.02E+05	2.09E+05	7.2%
Human.PERM.IANVFTNAFR.+2y5.light	Area	3.51E+04	3.75E+04	4.37E+04	3.92E+04	4.05E+04	8.3%		Area	3.84E+04	3.84E+04	3.67E+04	2.97E+04	3.00E+04	3.15E+04	12.1%
Human.PERM.IANVFTNAFR.+2y5.light	Area	1.00E+05	9.97E+04	1.04E+05	1.11E+05	9.83E+04	5.1%		Area	9.74E+04	7.80E+04	9.33E+04	8.24E+04	8.31E+04	9.20E+04	8.7%

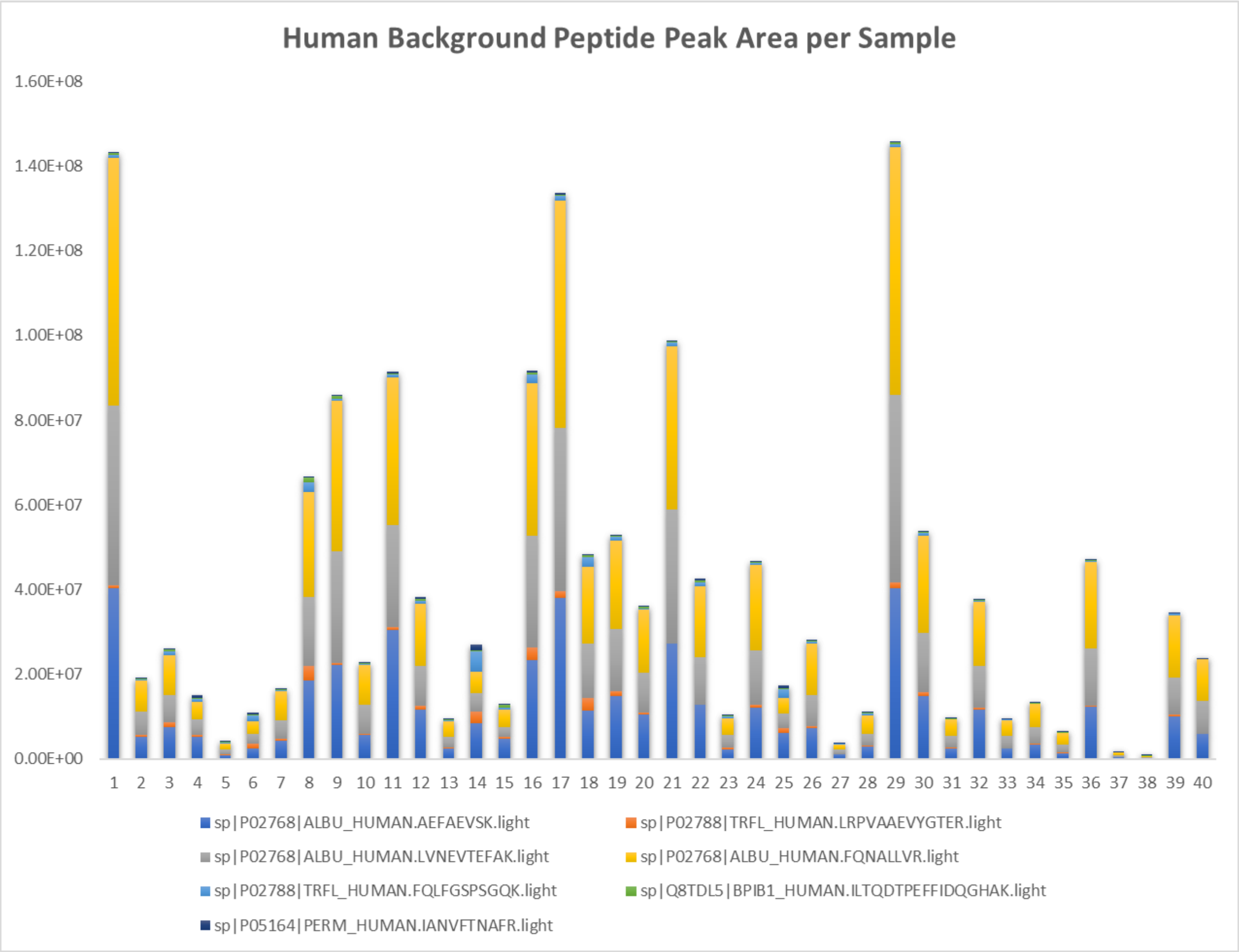

Figure S2. Background Protein Monitoring: plot of raw peak areas for seven peptides monitored in clinical specimens showing variability of human protein background.
